# High-efficiency protein expression in plants from agroinfection-compatible *Tobacco mosaic virus *expression vectors

**DOI:** 10.1186/1472-6750-7-52

**Published:** 2007-08-27

**Authors:** John A Lindbo

**Affiliations:** 1 Department of Plant Pathology, Ohio State University/OARDC, 1680 Madison Ave, Wooster, OH 44691, USA

## Abstract

**Background:**

Plants are increasingly being examined as alternative recombinant protein expression systems. Recombinant protein expression levels in plants from *Tobacco mosaic virus *(TMV)-based vectors are much higher than those possible from plant promoters. However the common TMV expression vectors are costly, and at times technically challenging, to work with. Therefore it was a goal to develop TMV expression vectors that express high levels of recombinant protein and are easier, more reliable, and more cost-effective to use.

**Results:**

We have constructed a *Cauliflower mosaic virus *(CaMV) 35S promoter-driven TMV expression vector that can be delivered as a T-DNA to plant cells by *Agrobacterium tumefaciens*. Co-introduction (by agroinfiltration) of this T-DNA along with a 35S promoter driven gene for the RNA silencing suppressor P19, from *Tomato bushy stunt virus *(TBSV) resulted in essentially complete infection of the infiltrated plant tissue with the TMV vector by 4 days post infiltration (DPI). The TMV vector produced between 600 and 1200 micrograms of recombinant protein per gram of infiltrated tissue by 6 DPI. Similar levels of recombinant protein were detected in systemically infected plant tissue 10–14 DPI. These expression levels were 10 to 25 times higher than the most efficient 35S promoter driven transient expression systems described to date.

**Conclusion:**

These modifications to the TMV-based expression vector system have made TMV vectors an easier, more reliable and more cost-effective way to produce recombinant proteins in plants. These improvements should facilitate the production of recombinant proteins in plants for both research and product development purposes. The vector should be especially useful in high-throughput experiments.

## Background

Plants offer great potential as production systems for recombinant proteins. One approach to producing foreign proteins in plants is to generate stable transgenic plant lines. However this is a very time consuming and labor intensive process. An alternative to transgenic plants is the use of plant virus-based expression vectors. Plant virus-based vectors allow for the rapid, high level, transient expression of proteins in whole plants (Review [1,2]). Although many different plant viruses have been modified to function as expression vectors, *Tobacco mosaic virus *(TMV)-based vectors express the highest levels of foreign protein in plants (Review [3,4]). TMV's utility as an expression vector has already been well established; TMV vectors have been used to produce of many different kinds of proteins in plants including allergens [5,6], antibodies [7] or antibody fragments [8], and vaccine candidates [9,10].

TMV is an RNA virus that expresses large amounts of coat protein from a viral subgenomic promoter. To convert TMV to an expression vector an additional, heterologous coat protein subgenomic promoter and restriction enzyme sites for cloning of foreign DNA sequences were inserted into a T7 promoter driven cDNA clone of TMV[11]. *In vitro *transcription of this plasmid with T7 RNA polymerase is needed to generate biologically active transcripts. Transcripts are typically rub-inoculated by hand onto plants to initiate an infection [4]. The *in vitro *transcription and rub inoculation steps in particular, add significantly to the cost and complexity of using TMV vectors.

Agroinfection [12,13] is a less-expensive and more reproducible strategy for infecting plants with RNA viruses. In agroinfection a plant-functional promoter and RNA virus cDNA are transferred as T-DNA from *Agrobacterium tumefaciens *into plant cells. The T-DNA is transcribed *in planta*, to generate biologically active viral RNAs that can initiate self-replication. Although agroinfection has been used for several different plant RNA viruses, it has not been routinely used with TMV-based vectors.

Recently, an agroinfection-compatible TMV replicon was constructed with extensive modifications to the TMV cDNA. These modifications included (1) the deletion of the TMV coat protein (CP) gene, (2) generating nearly 100 point mutations to destroy cryptic introns in the viral cDNA, and (3) inserting multiple (up to 24) plant-gene introns into the TMV cDNA sequences in a binary vector [14].

The point mutations and inserted introns dramatically improved the infectivity of the TMV replicon delivered to plants by agroinfection. However, because of the deletion of the CP gene, this replicon cannot move systemically in plants. Plants were therefore infected in a process called "magnifection" [9,15] in which whole plants were submerged and infiltrated with *A. tumefaciens *cultures carrying intron-modified TMV sequences in a binary vector. While the magnifection process is very efficient, it is not easily adapted to a high throughput workflow. Also, the increased size of the intron-modified vectors can make cloning into these vectors more challenging. In addition, it is not clear if the intron-modified vectors are absolutely required for efficient agroinfection of local and systemic infection of plant tissue with a TMV vector.

Therefore, there is a need for TMV expression vectors with the following features: 1) contains convenient cloning sites for genes of interest 2) can be used to infect plants in an easy and cost-effective manner, and 3) leads to efficient local and systemic infection of inoculated plants. To address these needs, a 35S promoter driven version of a commonly used TMV expression vector (containing the TMV CP gene) [11] was constructed in the T-DNA region of the mini-binary vector pCB301 [16]. This plasmid was then modified to contain restriction endonuclease sites for standard restriction endonuclease based cloning approaches or two SapI (type IIS) restriction endonuclease sites that are useful for a new, rapid, directional cloning strategy. In this new cloning strategy, referred to here as "sticky RICE" for Restriction enzyme-Independent Cohesive Ends, PCR products were directly cloned into a TMV vector, without the need for digesting PCR products with restriction enzymes.

The various 35S promoter-driven TMV vectors constructed reliably infected *Nicotiana benthamiana *plants via agroinfection. Using GFP-expressing TMV vectors, it was determined that the agroinfection efficiency of 35S driven TMV clones was significantly increased by ectopic expression of a protein with RNA silencing suppressor ability. This high-efficiency agroinfection procedure made it possible to express approximately 600 to 1200 μg of recombinant GFP per gram of agroinfiltrated tissue, and to reliably obtain plants that expressed GFP in systemically infected tissue. These results significantly extend the utility of the TMV expression vector system. The cloning and/or agroinfection procedure modifications described here can be readily applied to other cloning and expression vector systems and are especially amenable to high-throughput cloning workflows. These improvements should make it easier and more cost effective to use TMV-based plant expression vectors in research and development.

## Results

### Infecting plants with TMV vectors by agroinfiltration

**Figure 1:**
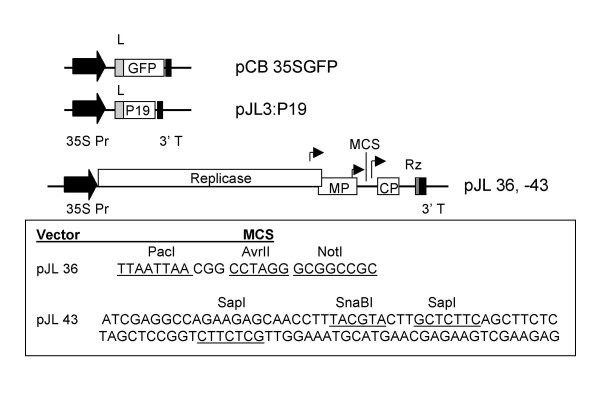
**Maps of plasmid T-DNAs used in this study**. *Cauliflower mosaic virus *(CaMV) 35S promoter driven versions of the *gfp *gene, *Tomato bushy stunt virus p19 *gene or *Tobacco mosaic virus *(TMV) vector cDNAs were constructed. All plasmids were based on the binary vector pCB301 backbone. T-DNA border sequences not shown in maps. Open boxes represent open reading frames; black block arrows; 35S promoter (35S Pr); black boxes, CaMV 3' terminator sequence; light grey boxes, *Tobacco etch virus *5' non-translated leader sequence (L); dark grey box, ribozyme (Rz); bent arrows, locations of TMV subgenomic promoters. The TMV vectors in pJL36 and pJL43 contain the full complement of TMV genes as well as an additional subgenomic promoter. TMV sequences 5' of the multiple cloning site (MCS) are from the U1 strain of TMV. Virus sequences 3' of the MCS are from the U5 strain of TMV. TMV transcripts are processed by a ribozyme to generate authentic TMV 3' ends. The sequence of the MCS in pJL 36 and 43 are presented. Restriction endonuclease recognition sequences are underlined. Because SapI is non-palindromic (GCTCTTC N1/4) both strands of the MCS of pJL 43 are presented for clarity.

**Figure 2:**
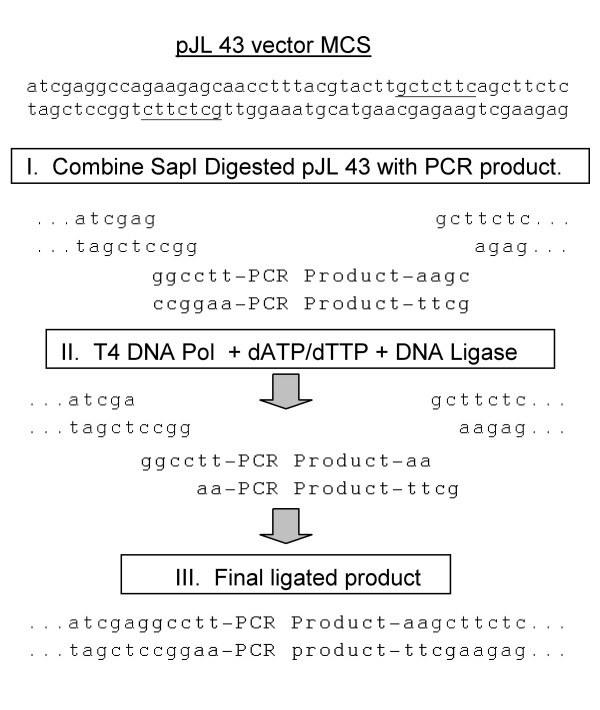
**Diagram of a Sticky RICE cloning reaction into pJL 43**. Sticky RICE cloning used a mixture of DNA polymerase and ligase (and, optionally, polynucleotide kinase) with specially designed vector and insert (PCR product) DNAs to directionally ligate DNAs. Single stranded 3 nt, 5' overhangs were generated on pJL 43 by digestion the restriction endonuclease SapI (underlined). Vector was treated with phosphatase after digestion to remove phosphates from 5' ends of DNA. I. Purified PCR product [amplified with 5' phosphorylated primers that began with 5'GGCCWW and 5'GCWW (W = A or T)] was added to SapI cut pJL 43. II. A mixture of T4 DNA polymerase, the nucleotides dATP/dTTP and T4 DNA ligase were added to combined vector and PCR product. During this step the 5' overhangs of SapI cut pJL 43 were altered by the T4 DNA polymerase. A single G residue was removed from the 3' end of the left end of the SapI cut vector, to generate a 5' overhang of GGCC. A single A residue was added to the 3' end of the right end of the SapI cut vector, to generate a 5' overhang of GC. Similarly, the 3' to 5' exonuclease activity of T4 DNA polymerase in the presence of dATP and dTTP removed G or C residues from the 3' ends of the PCR product. Complementary 5' overhangs in vector and PCR product (insert) guided annealing of DNAs. III. Annealed DNAs were joined by T4 DNA ligase. Sequences of the PCR product are in bold type. Vector sequences in final joined product are in all caps. The recognition sequences for the restriction endonucleases StuI (AGGCCT) and Hind III (AAGCTT) were generated at the vector-insert junctions.

A series of plasmids containing CaMV 35S promoter (35S) driven TMV vectors, as well as 35S driven versions of the green fluorescent protein (*gfp*) gene and the *p19 *(RNA silencing suppressor) gene from TBSV were constructed for this experiment (Figure 1). One of the TMV vectors, pJL 36, contained unique recognition sites for the restriction endonucleases PacI (TTAATTAA), AvrII (CCTAGG) and NotI (GCGGCCGC). This vector was useful for standard restriction enzyme based cloning procedures (data not shown). A second vector, pJL43, had a multiple cloning site that contained two SapI restriction endonuclease sites. The *gfp *gene was cloned into SapI digested pJL43 using the 'sticky RICE' procedure to generate plasmid pJL43:GFP. In this procedure 5' single stranded ends, complementary to the 5' single stranded ends on SapI digested pJL 43, were generated by treating specially engineered PCR products with T4 DNA polymerase in the presence of dATP and dTTP. The steps of the process are outlined in Figure 2. The sticky RICE cloning reaction could be completed in 30 minutes and resulted in approximately 90% cloning efficiency; 7 out of 8 clones recovered contained the *gfp *insert (Table 1).

**Table 1:** Cloning efficiency when using Sticky RICE method

**Insert size (bp)**	**5'PO_4 _on insert**	**Enzyme mix^a^**	**Reaction time**	**# of colonies**	**% with insert^b^**
770	No	Pol/Kin/Lig	30 min	77	~90%
770	Yes	Pol/Lig	30 min	365	~90%

^a^Pol, 0.25 Units (U) T4 DNA polymerase; Kin, 0.5 U T4 polynucleotide kinase; Lig, 400 U T4 DNA ligase.

^b^determined by restriction endonuclease screening of plasmids (n = 8)

To test the infectivity of the 35S driven TMV vectors, cultures of the GV3101 strain of *A. tumefaciens *(*A.t.*) transformed with pJL43:GFP were prepared essentially as described in Johansen and Carrington [17] and infiltrated into 2–4 week old *N. benthamiana *plants with a 1 ml syringe with no needle.

**Figure 3:**
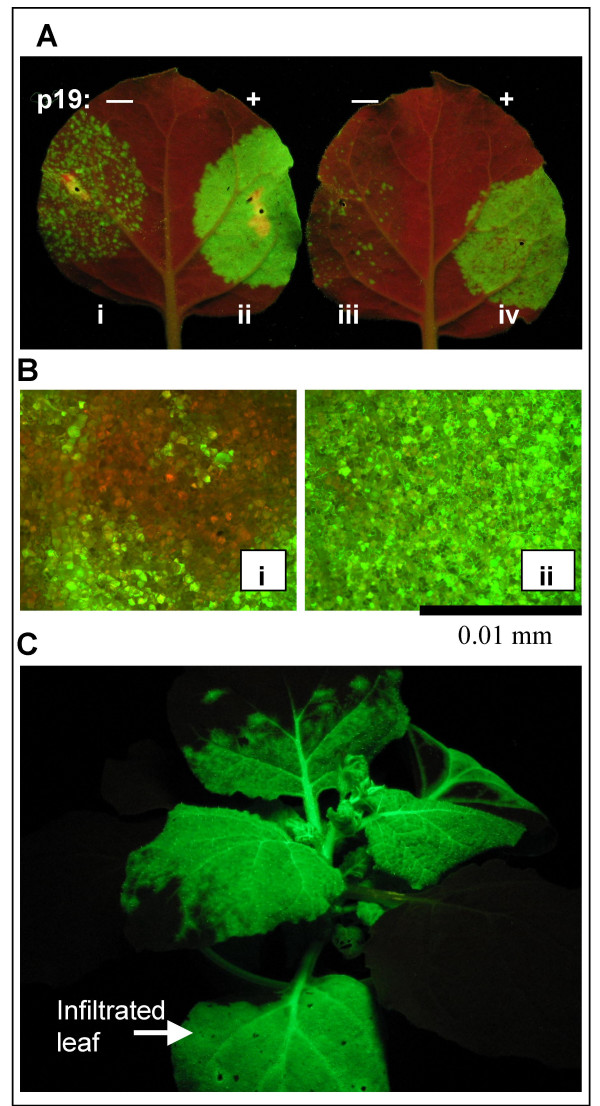
**Effect of *p19 *on the agroinfection efficiency of a *Tobacco mosaic virus*-based vector**. *N. benthamiana *leaves were infiltrated with mixtures of *A. tumefaciens *(*A.t.*) cells (OD_600 _1.0, or dilutions thereof) containing the binary plasmid pJL43:GFP and, in some treatments, pJL3:P19. pJL43:GFP has as its T-DNA a 35S promoter driven TMV vector with a green fluorescent protein (*gfp*) gene insert. When TMV RNA is transcribed from the T-DNA, the TMV vector initiates self-replication and expresses GFP. The GFP-expressing TMV vector can move cell-to-cell and systemically in plants. Photographs taken under UV light, where GFP appears green and non-GFP expressing tissue red. A. Leaves infiltrated with (i) 1:1 mix of *A.t.*/pJL43:GFP + *A.t.*/pJL4 (empty vector); (ii) 1:1 mix of *A.t.*/pJL43:GFP + *A.t.*/pJL3:p19; (iii) 1:1 mix of 1:50 dil of *A.t.*/pJL43:GFP + undiluted *A.t.*/pJL4; (iv)1:1 mix of 1:50 dil of *A.t.*/pJL43:GFP + undiluted *A.t.*/pJL3:p19. Photographed 3 days post infiltration (DPI). B. Fluorescent micrographs of infiltrated leaves, 3 DPI. Labelling same as in A. C. Systemic movement of TMV:GFP in *N. benthamiana *plant where a lower leaf was infiltrated with *A.t.*/pJL43:GFP cells. Photo taken 11 DPI.

Three days after infiltrating *A.t.*/pJL43:GFP cells into *N. benthamiana *leaves GFP-expressing cells could be seen using a hand-held UV lamp (Figure 3). Each GFP-positive cluster of cells represents a single incident of the GFP-expressing TMV vector RNA being "launched" from the transcribed T-DNA, and the TMV:GFP vector moving from the initially infected cell to adjacent cells. Hundreds of GFP-positive spots were detected per 10 cm^2 ^of infiltrated leaf (Figure 3A,i).

Because the agroinfection efficiency of a *Beet yellows virus *based vector was significantly increased by RNA silencing suppressor proteins [18] we sought to determine if RNA silencing suppressor proteins could increase the agroinfection efficiency of our TMV clones. To test this *N. benthamiana *plants were infiltrated with a 1:1 mixture of *A.t.*/pJL3:p19 and *A.t.*/pJL43:GFP cultures. When plants were infiltrated with the mixture of these two cultures a dramatic increase in the number of GFP-expressing cells was observed (Figure 3Aii). By three DPI it appeared that nearly 100% of the cells in the infiltrated tissue were infected with TMV:GFP (Figure 3Aii). To better demonstrate the effect of P19 on agroinfection efficiency, plants were infiltrated with a 50-fold dilution of *A.t.*/pJL43:GFP cells (diluted from an OD_600 _of 1.0), with and without co-infiltration of a *A.t.*/pJL3:p19 cells. Because a 50-fold dilution of A.t./pJL43:GFP co-infiltrated with A.t./pJL3:P19 (Figure 3A,iv) resulted in more than twice the number of GFP-positive foci per cm^2 ^of infiltrated tissue than infiltration with undiluted A.t./pJL43:GFP alone (Figure 3A,i) we estimated that ectopic expression of pJL3:P19 increased the agroinfectivity of our TMV expression vector construct by at least 100 fold. Efforts to further quantitate the effect on infectivity were not made.

After the TMV vector is launched it begins to move cell-to-cell and systemically in the plant. By about 7 DPI non-infiltrated (non-inoculated) leaves of the plant began to be infected by the viral vector. By about 11 DPI the systemic infection was well established, and very little non-GFP expressing systemic plant tissue was observed. The image in Figure 3C represents a typical *N. benthamiana *plant in which one leaf was co-infiltrated with *A.t.*/pJL43:GFP and *A.t.*/pJL3:P19 cultures. The photo was taken 12 DPI under UV illumination. Similar results were obtained with leaves infiltrated with *A.t.*/pJL36:GFP cell suspensions (data not shown). pJL36:GFP contains the GFP gene cloned into the PacI and AvrII sites of pJL36.

### Temporal analysis of recombinant protein expression from a TMV vector

**Figure 4:**
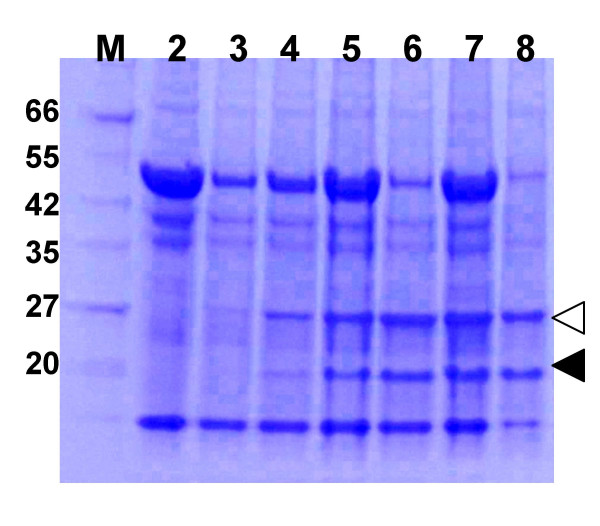
**Temporal analysis of GFP expression from TMV vectors**. *N. benthamiana *leaves were infiltrated with an *A. tumefaciens*/pJL43:GFP cell suspension. Total soluble protein extracts were prepared from infiltrated leaf tissue from 3–7 days post infiltration (DPI) or from plant tissue systemically infected with the TMV:GFP vector (12 DPI). Extracts were separated on a 4–20% SDS PAGE gel and stained with coomassie blue. Lanes: M, MW marker; 2, Healthy leaf extract; 3–7, extracts from *A. tumefaciens/*pJL43:GFP infiltrated leaves, 3–7 DPI, respectively; 8, extract from systemically infected tissue, 12 DPI. Marker band sizes (in kDa) are listed. Locations of GFP and TMV CP are identified by open and solid triangles, respectively.

To better examine the kinetics of recombinant protein expression from the TMV vector we prepared total soluble protein extracts from *A. tumefaciens*/pJL43:GFP + *A*. *tumefaciens*/pJL3:P19 infiltrated tissue from 3–7 DPI. Protein samples were also prepared from systemically infected plant tissue (12 DPI), to allow comparison of the amount of GFP produced in infiltrated vs. systemically infected plant tissue. Protein extract samples were separated on SDS PAGE gels and stained with coomassie blue protein stain. Results are shown in Figure 4. Recombinant GFP expression in infiltrated tissue increased up to about 4–6 dpi. Also the maximal GFP expression level observed in infiltrated tissue was comparable to the levels obtained in systemically infected leaves.

### Comparison of TMV expression vector with other transient expression systems

**Figure 5:**
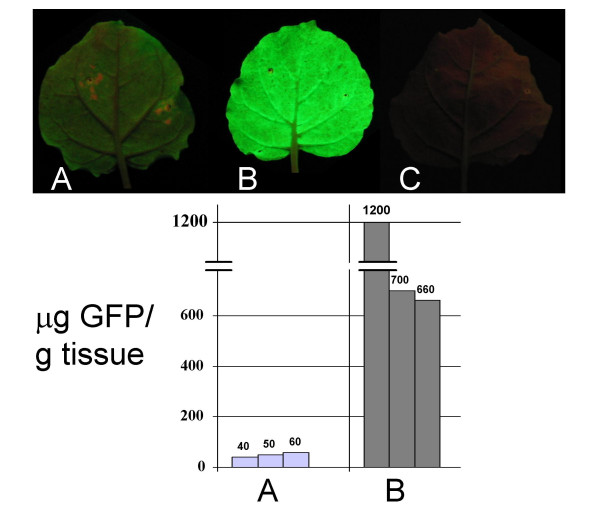
**Quantitation of transient expression of GFP in plants from 35S promoter or from a TMV vector**. Top. Individual *N. benthamiana *leaves infiltrated with (A) *A. tumefaciens (A.t.)*/pCB:GFP + *A.t.*/pJL3:P19 cell suspensions or (B) *A.t.*pJL43:GFP + *A.t.*/pJL3:P19 cell suspensions. (C) Non-infiltrated control leaf. Leaves photographed (4 days post infiltration, DPI) under UV light to visualize GFP. Bottom. Micrograms of GFP produced per gram of infiltrated tissue as estimated by GFP fluorescence assay. Labelling same as in top of figure. Plant extracts prepared 4–5 DPI were analyzed with a plate-based GFP fluorescence assay. Three plants of each treatment were analyzed. Samples were analyzed in triplicate, and values averaged. Purified His-6 tagged GFP was used to generate a standard curve.

An 'enhanced' agrobacterium mediated transient expression system has been described in which a 35S promoter driven gene-of-interest is co-introduced into cells along with a 35S driven *p19 *gene from TBSV [19]. The ectopic expression of the RNA silencing suppressor P19 dramatically enhanced the amount of recombinant protein produced from the 35S: gene-of-interest T-DNA. As a result, this approach is perhaps the most common method for transiently expressing proteins in plants. Therefore we sought to compare the relative expression efficiencies of this method with the TMV vector we constructed. *A. tumefaciens *cultures with plasmids pCB:GFP or pJL3:P19 were mixed and co-infiltrated into *N. benthamiana *leaves (Figure 5). Other leaves were co-infiltrated with a mixture of *A. tumefaciens *cultures containing pJL3:P19 or pJL43:GFP plasmids (Figure 5). Total soluble protein was extracted from infiltrated leaf tissue at 5 days post infiltration. The levels of GFP in extracts was determined by a plate-based fluorescence activity assay. The results revealed that the TMV based expression vector produced from 10 to 25 times more GFP than was expressed from 35S promoter driven *gfp *T-DNA co-infiltrated with the 35S driven *p19 *gene (Figure 5).

## Discussion

Recently it has been demonstrated that ectopic expression of proteins with RNA silencing suppressor activity increased (to varying degrees) the agroinfection rates of BYV[18] and CMV [20] vectors. Here we describe the construction of a new TMV vector that can be delivered by agroinfection and demonstrate that the RNA silencing suppressor protein P19 from TBSV can improve the agroinfectivity of TMV-based expression vectors. The agroinfection rate of the 35S driven TMV vector has been increased so much that coomassie blue stainable levels of TMV-expressed recombinant protein can be detected in crude protein extracts from agroinfiltrated tissue approximately 4 days post infiltration. This improvement eliminates the need for waiting for the TMV vector to move systemically in the inoculated plants.

Because TMV is a rod-shaped virus, there is no theoretical limit on the size of the foreign gene that can be inserted into a TMV vector. However, in practice, when plant virus vectors containing foreign inserts move systemically in plants they can lose the foreign insert. The larger the insert the more likely that insert loss will be a problem [21]. The ability to purify recombinant proteins from infiltrated tissue rather than tissue systemically infected by a viral vector is expected allow larger proteins to be reliably expressed from TMV vectors without concerns about insert stability in the virus. Furthermore we demonstrate that our *A. tumefaciens *delivered TMV vector, produced from 10 to 25 times more GFP than was obtained from the 'enhanced' agrobacterium transient expression system in which both *gfp *and *p19 *are co-expressed from 35S promoters [22].

The efficiency of TMV vector agroinfection we have obtained (through the co-introduction of a 35S driven RNA silencing suppressor gene) appears to be comparable to the TMV vector agroinfection efficiencies recently reported by others [14]. However, it is important to note their vector lacked the TMV CP gene, and was therefore not capable of moving systemically in plants. In their approach to improving TMV vector agroinfection rates, Marillonnet et al [14] both removed potential cryptic introns (by making nearly 100 silent mutations to the virus cDNA sequence) and inserted introns into the viral cDNA. Their final optimized construct had multiple silent mutations and 16 introns inserted into the viral cDNA [14]. In this paper we demonstrate that it is possible to dramatically improve agroinfection efficiency of a TMV vector without destroying cryptic introns or introducing foreign introns into the TMV cDNA. It is interesting that these two different approaches could both be used to improve the agroinfectivity of a TMV vector. One explanation of this phenomenon is that these two approaches are mechanistically related, as discussed below.

In our current model we hypothesize that wild-type TMV RNA generated from a T-DNA in a plant nucleus is often spliced resulting in the removal of critical viral RNA sequences from the genome. As a result TMV RNAs that are not capable of self-replication are often exported to the plant cytoplasm where they are processed by the cells RNA silencing machinery. RNA silencing (also called RNA interference or RNAi) is a well-reviewed topic (Review [23-25]). Briefly, in plants small RNAs of about 21 nts in length associate with a protein complex called RISC (RNA induced silencing complex) that has nuclease activity. The small RNAs associated with RISC serve to guide the nuclease to specific RNA targets through base-pairing whereupon the target RNA is cleaved. The small "guide" RNAs are generated by cellular dicer-like enzymes, that cleave ds RNAs into duplexes of about 21 nt. The TMV-derived RNAs exported from the plant nucleus in our system may serve as dicer substrates either because they have regions of secondary structure, and/or the RNAs are converted to dsRNAs by the activity of cellular RNA dependent RNA polymerases [26]. Regardless of the exact pathway our model proposes that short RNAs (~21 nt in length) produced primarily from defective TMV RNA sequences are used to program the cellular RISC (RNA induced silencing complex) to target TMV RNAs for degradation. As a result when the occasional full-length TMV RNA, which was fortuitously not spliced at cryptic introns, was exported from the nucleus into the cytoplasm it was degraded by a TMV-specific RISC.

When *p19 *is expressed in the plant cell the P19 protein binds 21 nt duplex RNAs [27-29], preventing them from being incorporated into RISCs. As a result in these plant cells when the occasional full length TMV RNA transcript does enter the cytoplasm it has a greater potential to be translated and initiate self-replication before it is degraded. It is proposed that this is why the P19 protein enhances agroinfection efficiency of a wild-type TMV cDNA.

In contrast, the intron-modified TMV cDNA generated by Marillonet et al [14] must have resulted in a higher percentage of replication-competent TMV RNAs being exported into the cytoplasm. As a result TMV self-replication was often established with this vector before the TMV-targeted RISC was generated in the plant. In this model these two approaches for enhancing the agroinfectivity of a TMV cDNA are complementary. One would therefore predict that the two methods could be combined to obtain even higher agroinfection rates of the 35S-driven TMV clones. Also this model would predict that agroinfection rates of 35S promoter driven TMV (non-intron modified) cDNAs would be higher in plants mutant for certain genes in the RNA silencing pathway, and that the ectopic expression of multiple, different, RNA silencing suppressor proteins may further increase agroinfectivity of these vectors.

## Conclusion

A simple, cost effective, and efficient way to infect plants with TMV vectors via agroinfection was identified. Recombinant protein expression levels of approximately 600 to 1200 micrograms per gram of *A. tumefaciens *infiltrated plant tissue were obtained. Recombinant protein can be recovered from both locally inoculated leaves or from systemically infected plant tissue. These improvements should enable researchers with little or no experience with plant virus vectors to easily utilize TMV expression vectors in their research. The vector improvements described here will be especially useful in high-throughput research projects.

## Methods

### PCR Reactions

Taq DNA polymerase (New England Biolabs) was used for all PCR reactions, using manufacturers instructions. For production of 5' phosphorylated PCR products, both forward and reverse direction PCR primers were treated with T4 polynucleotide kinase (New England Biolabs) according to manufacturers instructions, prior to use in the PCR. Amplified reaction products were column purified with the DNA clean up and concentrator kit (Zymoresearch) to remove unincorporated dNTPs and primers.

### Plasmid construction

The duplicated CaMV 35S promoter, the *Tobacco etch virus *(TEV) 5' non-translated leader sequence, a short polylinker containing an XbaI site, and the polyA/terminator sequences from pRTL2 [30] were cloned as a PstI fragment into PstI cut binary vector pCB301 [16] to generate pJL3. Using inverse PCR a PacI restriction endonuclease site was generated immediately downstream of the TEV leader sequence. The *gfp *or *p19 *genes were cloned into the PacI and XbaI sites of the vector to generate pCB35SGFP and pJL3:P19, respectively. The "cycle 3" mutant version of *gfp *[31] in p30BGFP [11] was used as the source of the *gfp *gene. The coding sequence for the *p19 *gene from TBSV was amplified from a cDNA sample (a kind gift from H. Scholthof).

The NotI restriction endonuclease site in the pCB301 backbone of pCB35S:GFP was destroyed by digesting with NotI, treatment with T4 DNA polymerase and dNTPs followed by religation to generate pCB 35SGFP ΔN. Inverse PCR of pCB 35SGFP ΔN was used to generate a unique StuI restriction site at the 35S promoter transcription start site as described in Dessens and Lomonossoff [32]. The resulting plasmid was named pJL 22.

The TMV expression vector in p30B [11] contains a T7 driven cDNA of the U1 strain of TMV, an additional viral subgenomic promoter for expression of foreign gene inserts, and a ribozyme sequence following the end of the viral cDNA (Figure 1). Using standard cloning procedures the cDNA version of the TMV expression vector and ribozyme sequence from p30B (obtained from Large Scale Biology Corporation, Vacaville, CA) was cloned into StuI-XbaI cut pJL 22. This plasmid was then modified to generate pJL36 and pJL43. In pJL36 the multiple cloning site (seq ttaattaacggcctagggcggccgc) was inserted downstream of the additional TMV subgenomic promoter. pJL36, (Figure 1) has unique PacI, AvrII and NotI restriction endonuclease sites for cloning.

To construct pJL43 (Figure 1) the ds DNA cassette [top stand seq (CGAGGCCAGAAGAGCAACCTTTACGTACTTGCTCTTCAGCTTGAAGGTAAGCCTATCCCTAACCCTCTCCTCGGTCTCGATTCTACGCGTACCGGTCATCATCACCATCACCATTGAC)] containing the coding sequence for two SapI restriction endonuclease sites (gctcttc), the V5 epitope (amino acid sequence GKPIPNPLLGLD) and a hexa-histidine (His6) tag coding sequence was inserted downstream of the additional TMV subgenomic promoter.

### Sticky RICE cloning into pJL 43

#### Preparation of vector

To prepare pJL-43 for sticky RICE cloning, plasmid DNA was digested with the restriction endonuclease SapI, treated with calf alkaline intestinal phosphatase and column purified.

#### Preparation of PCR products

For sticky RICE cloning of PCR products into SapI cut pJL 43 the 5' end of the forward PCR direction primer must have the following sequence: 5' GGCCWW. The 5' end of the reverse direction PCR primer should be 5' GCWW. In the primer sequences W = A or T. The T and A residues in the primers will serve as "stop nucleotides" during the sticky RICE reaction, in which PCR products were incubated in the presence of a DNA polymerase with 3' to 5' exonuclease activity and dATP and dTTP. The DNA polymerase removed nucleotides from the 3' ends of each strand until an A or T residue was present in the template stand. This resulted in 5' overhangs on each end of the PCR products (Figure 2).

Using these guidelines for primer design, the *gfp *gene was amplified by the PCR from a pUC-based plasmid with forward direction primer JAL 286 (**GGCC**T aaa atggctagcaaaggagaag) and reverse direction primer JAL 287 (**GC**ttatttgtagagctcatccat). The primer nucleotides that were converted to sticky ends by T4 DNA polymerase are in bold. PCR reactions using either non-phosphorylated or 5' phosphorylated primers were performed. PCR products were purified to remove unincorporated dNTPs and primers and eluted in dH_2_0.

#### Assembly of the Sticky RICE reaction

A three-fold molar excess of phosphorylated *gfp *gene PCR product was combined with 50 ng SapI cut and phosphatase treated pJL 43 DNA in a 10 ul reaction volume composed of: 1 × ligase buffer, 0.1 mM (each) dATP/dTTP, 0.25 Units T4 DNA Polymerase, and 400 Units T4 DNA ligase. Enzymes, buffers and unit definitions were all from New England Biolabs. The assembled reaction was incubated at room temperature for 30 minutes.

For purposes of comparison, non-phosphorylated *gfp *gene PCR products were also cloned using the same reaction conditions except that 0.5 Units T4 polynucleotide kinase were also included in the Sticky RICE cloning reaction. The cloning of the *gfp *gene into pJL 43 resulted in plasmid pJL43:GFP.

### Transformation of E. coli with Sticky RICE cloning reactions

Two microliters of a sticky RICE ligation reaction was added to 25 ul of chemically competent *E. coli *(Bioline, AlphaSelect Gold efficiency). Transformation conditions were essentially as per manufacturers instructions. Transformed cells were plated on LB plates with 50 ug/ml Kanamycin.

### Screening plasmids for inserts

Following transformation of the sticky RICE ligations into *E. coli*, individual colonies were used to inoculate LB broth with 50 ug/ml Kanamycin. Plasmids were purified from overnight liquid cultures. Because the forward-direction primer used for PCR began with the sequence "GGCCT" a StuI restriction enzyme recognition site was generated upon sticky RICE joining of PCR product to pJL 43. Therefore plasmids were screened by digestion with StuI and digested DNA separated on 1% agarose gels to identify clones that had inserts (Table 1).

### Agroinfection

Plasmids purified from *E. coli *cultures were transformed into *A. tumefaciens *GV3101 using the freeze thaw method [33]. Transformed *A. tumefaciens *were plated on LB plates with 50 ug/ml Kanamycin, 25 ug/ml gentamycin and 10 ug/ml rifampicin for plasmid selection. Individual colonies of *A. tumefaciens *transformed with a binary plasmid were grown to an OD_600 _of 1.0 in liquid LB media supplemented with 10 mM MES pH 5.7, 50 ug/ml Kanamycin, 25 ug/ml gentamycin and 20 uM acetosyringone. Cells were collected by centrifugation and resuspended in 10 mM MES, pH 5.7, 10 mM MgCl2, 200 uM acetosyringone [17]. Cells sat at room temperature in induction media for 2–24 hours before infiltration into the abaxial surface of *N. benthamiana *leaves using a 1 ml syringe with no needle. *A. tumefaciens *cultures containing an 'empty vector control' plasmid or pJL3:p19 were co-infiltrated with *A. tumefaciens*/pJL43:GFP cultures. When mixes of *A. tumefaciens *cultures were infiltrated, bacterial cultures were prepared separately in induction media and were combined immediately before infiltration.

### SDS-PAGE

Healthy and TMV:GFP infected plant tissue (from TMV vector launched from the *A*. *tumefaciens*-delivered T-DNA of pJL43:GFP) samples were ground in the presence of 4 volumes (per gram fresh weight) 50 mM Tris, pH 7.5, 150 mM NaCl, 0.1% Tween-20, 0.1% beta-mercaptoethanol. Extracts were clarified by centrifuging for 15 minutes at 12–15 K × g. Samples of clarified extract were combined with SDS-PAGE loading dye and analyzed on 4–20% SDS-PAGE gels. Gels were stained with coomassie blue to visualize proteins.

### GFP Assay

Plant protein extracts (prepared as described above) were diluted in 50 mM Carbonate buffer (pH 9.6). Samples were assayed for GFP activity with a Perkin-Elmer HTS 7000 BioAssay Reader using filters of 405 nm excitation/535 nm emission. A standard curve was prepared from purified His-6 tagged GFP (purified from plants).

## Abbreviations

TMV- Tobacco mosaic virus.

GFP- Green Fluorescent Protein.

MW- Molecular Weight.

DPI- Days Post Infiltration.

ds- Double stranded.

MP- Movement protein.

CP- Coat (capsid) protein.  

## Authors' contributions

JAL conceived of this study, designed and performed all experiments and wrote the manuscript. All authors read and approved the final manuscript.
